# Supplementary material for: Virological, Serological and Clinical Analysis of Chikungunya Virus Infection in Thai Patients
Source: Viruses. 2022 Aug 18;14(8):1805. doi: 10.3390/v14081805 (PMC9414365; doi:10.3390/v14081805)
Supplement: Supplementary file 1 [file viruses-14-01805-s001.zip › viruses-1832972-supplementary.pdf]

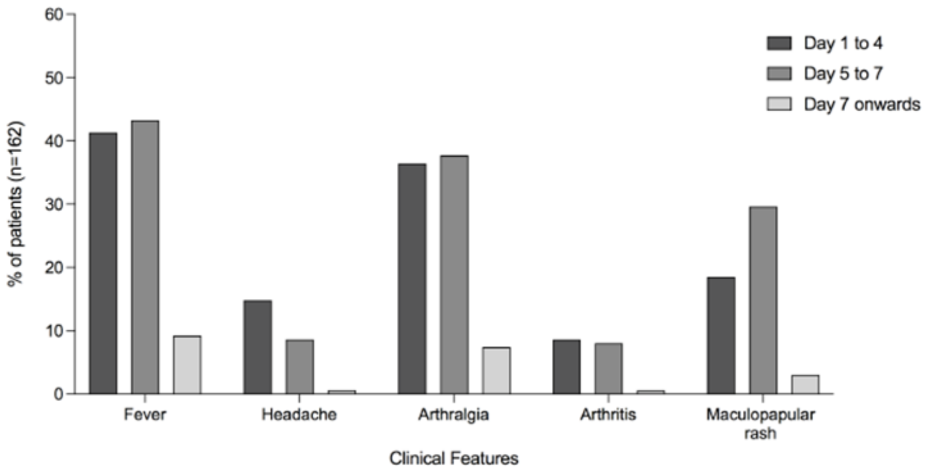

S1 Fig. Onset of symptoms during the acute phase of CHIKV infection.

S1 Table: CHIKV primers used in present study.

Primers	Region	Genome position	Sequence (5' → 3')
**Standard RT-PCR**			
CHIKV F	Nsp1	77-101	GCATTAAGCTTATGGATCCTGTGTACGTGGACATAG
CHIKV R		1658-1681	GCATTTCTAGATGCACCCGCTCTGTCCTCAAGCTG
**Real time RT-PCR**			
CHIKV Probe	Nsp1	201-224	TCCGACGTCATCCTCCTTGCTGGC
CHIKV F		165-185	TGATCCCGACTCAACCATCCT
CHIKV R		226-247	GGCAAACGCAGTGGTACTTCCT
**Sequencing**			
CHIKV 10123 F	E1	10123-10139	CGGCGCCTACTGCTTCTGCG
CHIKV 11287 R		11270-11287	CGACACGCATAGCACCAC
CHIKV 10014 F	E1	10014-10035	CCCGAACACGGTGGGAGTACCG
CHIKV 11227 R		11206-11227	TCCCGTGATCTTCTGCACCCAT

S2 Table: CHIKV sequences used in present study.

Strain	Genotype	Country	Accession Number
TM073	ECSA	Thailand	ON406423
TM217	ECSA	Thailand	ON406424
TM155	ECSA	Thailand	ON406425
TM009	ECSA	Thailand	ON406426
TM248	ECSA	Thailand	ON406427
TM004	ECSA	Thailand	ON406428
TM007	ECSA	Thailand	ON406429
TM008	ECSA	Thailand	ON406430
TM015	ECSA	Thailand	ON406431
TM025	ECSA	Thailand	ON406432
TM026	ECSA	Thailand	ON406433
TM033	ECSA	Thailand	ON406434
TM198	ECSA	Thailand	ON406435
